# Gene perturbation and intervention in context-sensitive stochastic Boolean networks

**DOI:** 10.1186/1752-0509-8-60

**Published:** 2014-05-21

**Authors:** Peican Zhu, Jinghang Liang, Jie Han

**Affiliations:** 1 Department of Electrical and Computer Engineering, University of Alberta, Edmonton, AB, Canada

**Keywords:** Gene regulatory networks, Boolean networks, Stochastic Boolean networks, Context dependent, Gene perturbation, Intervention, Context switch, Steady state distribution, p53 network, glioma network

## Abstract

**Background:**

In a gene regulatory network (GRN), gene expressions are affected by noise, and stochastic fluctuations exist in the interactions among genes. These stochastic interactions are context dependent, thus it becomes important to consider noise in a context-sensitive manner in a network model. As a logical model, context-sensitive probabilistic Boolean networks (CSPBNs) account for molecular and genetic noise in the temporal context of gene functions. In a CSPBN with *n* genes and *k* contexts, however, a computational complexity of *O*(*nk*^2^2^2*n*^) (or *O*(*nk*2^*n*^)) is required for an accurate (or approximate) computation of the state transition matrix (STM) of the size (2^*n*^ ∙ *k*) × (2^*n*^ ∙ *k*) (or 2^*n*^ × 2^*n*^). The evaluation of a steady state distribution (SSD) is more challenging. Recently, stochastic Boolean networks (SBNs) have been proposed as an efficient implementation of an instantaneous PBN.

**Results:**

The notion of stochastic Boolean networks (SBNs) is extended for the general model of PBNs, i.e., CSPBNs. This yields a novel structure of context-sensitive SBNs (CSSBNs) for modeling the stochasticity in a GRN. A CSSBN enables an efficient simulation of a CSPBN with a complexity of *O*(*nLk*2^*n*^) for computing the state transition matrix, where *L* is a factor related to the required sequence length in CSSBN for achieving a desired accuracy. A time-frame expanded CSSBN can further efficiently simulate the stationary behavior of a CSPBN and allow for a tunable tradeoff between accuracy and efficiency. The CSSBN approach is more efficient than an analytical method and more accurate than an approximate analysis.

**Conclusions:**

Context-sensitive stochastic Boolean networks (CSSBNs) are proposed as an efficient approach to modeling the effects of gene perturbation and intervention in gene regulatory networks. A CSSBN analysis provides biologically meaningful insights into the oscillatory dynamics of the p53-Mdm2 network in a context-switching environment. It is shown that random gene perturbation has a greater effect on the final distribution of the steady state of a network compared to context switching activities. The CSSBN approach can further predict the steady state distribution of a glioma network under gene intervention. Ultimately, this will help drug discovery and develop effective drug intervention strategies.

## Background

Diverse biological functions are regulated through the interactions among genes, proteins and other molecules in a cell. Gene expressions are however affected by the intrinsic and extrinsic noise in a gene network [1]. A major source of the noise is the stochastic fluctuations in gene regulatory interactions [2]. The genetic interactions are also context dependent, that is, certain regulatory functions are active in some cellular states, but inactive in others [3]. This indicates the necessity to consider noise in a context-sensitive manner in the study of gene regulatory networks (GRNs).

Various methods have been proposed to model GRNs; these include logical models [4], continuous models using differential equations [5,6] and stochastic models at the single-molecule level [7,8]. As a classic logical model, Boolean networks (BNs) have been widely used to qualitatively model the interactions among genes [4,9-11]. Probabilistic Boolean networks (PBNs) have been proposed to consider noise in a BN model [12-14]. In a PBN, the next state of a gene is determined by its current state and a Boolean function. If the Boolean function is randomly selected, a PBN is referred to as an instantaneous PBN [12]. As a general model, a context-sensitive PBN (CSPBN) considers the feature of context dependence in a biological network [15]. In a CSPBN, a context is a combination of Boolean functions and each function determines the next state of a gene. A context remains unchanged until a switching occurs. This switching of contexts, possibly caused by external stimuli, is considered to occur randomly in a network.

The study of PBNs has focused on the analysis of steady state distributions (SSDs) under gene perturbation and intervention. A Markov Chain Monte Carlo method is used in [16] to analyze the long run behavior of a PBN; however, this method generally requires a large number of simulations to reach a steady state, due to the slow convergence typically encountered in a Monte Carlo method [17]. An analysis is performed in [18] for finding the SSD of a PBN through the computation of the state transition matrix (STM). However, the application of an analytical approach is generally limited to small networks due to the exponential increase in the size of an STM with gene numbers. The analysis of CSPBNs presents even a greater challenge due to its significantly increased computational complexity. Analytical expressions have been derived for analyzing CSPBNs [19]; however, this method is only applicable to the steady state analysis of a network with small perturbation and switching probabilities. The method in [20] ignores some BNs with very small probabilities for reducing the size of the STM and thus provides a more efficient but approximate solution for computing the SSD of a CSPBN. In [15,21,22], gene intervention is investigated for avoiding undesirable states associated with certain diseases (such as cancer). Due to external stimuli, the STM is changed by external control variables, so desirable states can be obtained with larger probabilities in the SSD. In a context-sensitive network with *n* genes and *k* contexts, however, a (2^*n*^ ∙ *k*) × (2^*n*^ ∙ *k*) [23] (or 2^*n*^ × 2^*n*^[20]) matrix is required for an accurate (or approximate) analysis of the SSD; this results in a computational complexity of *O*(*nk*^2^2^2n^) (or *O*(*nk*2^*n*^)) for an accurate (or approximate) computation of the STM. Hence, the application of current gene network analysis is limited to those of less than a dozen of genes.

Recently, stochastic Boolean networks (SBNs) have been proposed for an efficient computation of the STM and SSD of an instantaneous PBN [24]. The SBN approach can recover biologically-proven regulatory behaviors, such as the oscillatory dynamics of a simplified p53-Mdm2 network [25] and the dynamic attractors in a T cell immune response network [26]. To further exploit the simplicity of logical models, stochastic multiple-valued networks (SMNs) have been utilized to investigate the dynamics of gene networks with multiple-valued gene states [27]. Asynchronous SBNs have also been developed to investigate the asynchronous state-update behavior of genes [28]. In this paper, the notion of SBNs is extended for the general model of PBNs, i.e., the CSPBNs. This presents a novel structure of context-sensitive SBNs (CSSBNs) for modeling the stochasticity in a context-dependent GRN. In particular, gene perturbation and intervention are considered in CSSBNs. Through an efficient simulation of a CSSBN, the computational complexity in the evaluation of a CSPBN is reduced from *O*(*nk*^2^2^2*n*^) to *O*(*nLk*2^*n*^) for computing the STM, where *L* is a factor related to the required sequence length in CSSBN for achieving a desired accuracy. The use of non-Bernoulli sequences of random permutations of fixed number of 1’s and 0’s further increases computational efficiency and allows for a tunable tradeoff between accuracy and efficiency. A time-frame expanded CSSBN can further simulate the stationary behavior of a CSPBN and produce results that are more accurate than an approximate analysis, thus making the CSSBN useful in modeling complex context-sensitive GRNs. The CSSBN models are applied to the study of a simplified p53-Mdm2 network and the results using an SSD control policy are reported on the effect of external gene intervention in a glioma network [18,20].

## Methods

### Context-Sensitive Probabilistic Boolean Networks (CSPBNs)

For a network of *n* genes, a probabilistic Boolean network (PBN) is defined by *G*(*V, F*), where *V* = {*x*_1_, *x*_2_, …, *x*_*n*_}, a set of binary-valued nodes, *F* = (*F*_1_, *F*_2_, …, *F*_*n*_), a list of sets of Boolean functions: $F_{i}=\left\{f_{1}^{\left(i\right)},f_{2}^{\left(i\right)},\ldots ,f_{l\left(i\right)}^{\left(i\right)}\right\}$ and *l*(*i*) is the number of possible functions for gene *i, i* = 1, 2, …, *n*[12-14]. A node *x*_*i*_ represents the state of gene *i*; *x*_*i*_ = 1 (or 0) indicates that gene *i* is (or not) expressed. The set *F*_*i*_ contains the rules that determine the next state of gene *i*. Each $f_{j\left(i\right)}^{\left(i\right)}:{\left\{0,1\right\}}^{n}\to \left\{0,1\right\}$ for 1 ≤ *j*(*i*) ≤ *l*(*i*) is a mapping or predictor function determining the state of gene *i*. In a context-sensitive PBN (CSPBN) with *k* contexts, a network function is defined as $f_{j}=\left(f_{j}^{\left(1\right)},f_{j}^{\left(2\right)},\ldots ,f_{j}^{\left(n\right)}\right)$, where $f_{j}^{\left(i\right)}:{\left\{0,1\right\}}^{n}\to \left\{0,1\right\}$ is the predictor function for gene *i, i* = 1, 2, …, *n,* in context *j* (*j* = 1, 2, …, *k*) [15,29].

Due to the stochasticity in genetic networks, the next state of gene *i* is determined by all the *l*(*i*) functions in *F*_*i*_, i.e., by $f_{1}^{\left(i\right)},f_{2}^{\left(i\right)},\ldots ,f_{l\left(i\right)}^{\left(i\right)}$, with probabilities $c_{1}^{\left(i\right)},c_{2}^{\left(i\right)},\ldots ,c_{l\left(i\right)}^{\left(i\right)}$. For an instantaneous PBN of *n* genes, there are a total number of $\prod_{i=1}^{n}l\left(i\right)$ possible Boolean networks (BNs), each of which is a possible realization of the genetic network. Each BN can be considered as the network function for a context [20]. In the *j*th context, assume the network function is given by $f_{j}=\left(f_{j\left(1\right)}^{\left(1\right)},f_{j\left(2\right)}^{\left(2\right)},\cdots ,f_{j\left(n\right)}^{\left(n\right)}\right)\;$, where each $f_{j\left(i\right)}^{\left(i\right)}:{\left\{0,1\right\}}^{n}\to \left\{0,1\right\}$ for 1 ≤ *j*(*i*) ≤ *l*(*i*) is a mapping or predictor function determining the state of gene *i*. The probability that the *j*th context is selected, is obtained as $C_{j}=\prod_{i=1}^{n}c_{j\left(i\right)}^{\left(i\right)}$ for *j* = 1, 2, …, *k*, where *k* is the number of contexts in a context-sensitive network [20]. The state of a gene is updated in the selected context; thus the next state depends on both the present state and the selected context.

In a context-sensitive network, a context may remain for certain time until a random event occurs. A context switching usually occurs with probability *q*. For *q* = 1, the CSPBN becomes an instantaneous PBN. For *q* < 1, if a new context is to be selected, it is randomly chosen from the set of network functions: {*f*_1_, *f*_2_, …, *f*_*k*_}, with a set of context selection probabilities: {*C*_1_, *C*_2_, …, *C*_*k*_} [29]. If noise is considered in a CSPBN, it is often referred to as a perturbation, by which a gene flips its state with a probability *p* (*p* ≠ 0). It has been shown that a PBN with perturbation is an ergodic Markov chain in that all the states are connected in the PBN [13]. The transition probability for any two states is determined by the values of *p* and *q* pairs. Following [19], one of four mutually exclusive events occurs at time *t* in a CSPBN with perturbation:

∅_1_: The predictor functions in the currently selected context are applied to update the gene expressions and this context remains for the next transition.

∅_2_: The predictor functions in the currently selected context are applied to update the states of the genes and then a new context is selected for the next transition.

∅_3_: A random perturbation occurs and the currently selected context remains for the next transition.

∅_4_: A random perturbation occurs and a new context is selected for the next transition.

The effect of a switching order, i.e., whether a network switches its context before or after its state transition, is considered in [23], whereas in this paper, we focus on the transition rules, i.e., the network function is applied first and then the context switches.

A gene activity profile (GAP) is defined as a vector for describing the state of a network at time *t*, ***x*** (*t*) = (*x*_1_(*t*), *x*_2_(*t*), …, *x*_*n*_(*t*)), where *x*_*i*_(*t*) ∈ {0, 1} for *i* = 1, 2, …, *n*. The state of a CSPBN can be represented as a combination of a context and a GAP, i.e., ***S*** = (*context j*, *GAP i*). By this definition, the number of states in a CSPBN increases from 2^*n*^ to 2^*n*^*k* for a network with *n* genes and *k* contexts. A GAP can also be represented by its decimal index, i.e., $d=\sum_{j=1}^{n}2^{n-j}x_{j}\left(t\right)+1$. A state of a CSPBN is then given by ***S*** = (***f***_*i*_, *d*) for *i* ∈ {1, 2, …, *k*} and *d* ∈ {1, 2, …, 2^*n*^}, where *d* is the decimal index of a GAP. For convenience, a state (***f***_*i*_, *d*) is referred to as (*i*, *d*) in the following analysis.

In a CSPBN with a perturbation probability *p* and a switching probability *q*, the transition probability for any two states ***a*** and ***b*** is given by [23]:

(1) $$\begin{array}{l} p\left(S_{t+1}=b|S_{t}=a\right)=\left\{{\left(1-p\right)}^{n}{f_{r_{1}}}_{{}_{,}x_{1},x_{2}}+{\left(1-p\right)}^{n-h}p^{h}s\left(h\right)\right\}\\ \;\cdot \left[\left(1-q+qc_{r_{1}}\right)g\left(a,b\right)+q{c_{r}}_{{}_{2}}\left(1-g\left(a,b\right)\right)\right] \end{array}$$

where

(2) $$f_{r_{1},x_{1},x_{2}}=\;\left\{\begin{array}{c} 1,\\ 0, \end{array}\begin{array}{c} \begin{array}{cc} & \;\mathrm{if}\;x_{1}\;\mathrm{directly}\;\mathrm{transitions}\;\mathrm{to}\;x_{2}\;\mathrm{in}\;\mathrm{context}\;r_{1} \end{array}\\ \begin{array}{cc} \begin{array}{cc} \begin{array}{cc} \begin{array}{cc} \begin{array}{cc} \begin{array}{cc} & \mathrm{otherwise} \end{array} & \end{array} & \end{array} & \end{array} & \end{array} & \end{array} \end{array}\right)$$

(3) $$g\left(a,b\right)=\left\{\begin{array}{c} 1,\\ 0, \end{array}\begin{array}{c} \begin{array}{ccc} & \mathrm{if}\;r_{1}=r_{2}=r & \end{array}\\ \mathrm{otherwise} \end{array}\right)\;$$

(4) $$s\left(h\right)=\left\{\begin{array}{c} 0,\\ 1, \end{array}\begin{array}{c} \;\mathrm{if}\;h=0\\ \;\mathrm{otherwise} \end{array}\right)\;$$

and *h* is the Hamming distance between two GAPs with decimal indices *x*_1_ and *x*_2_, *x*_1_, *x*_2_ ∈ {1, 2, …, 2^*n*^}. This distance indicates the number of genes with different expressions in the two GAPs *x*_1_ and *x*_2_.

The state transition matrix (STM) of a CSPBN without perturbation is of the size 2^*n*^ ∙ *k* × 2^*n*^ ∙ *k*, given by [23]:

(5) $$A=\left[\begin{array}{c} \left(1-q+qc_{1}\right)P_{1}\\ \cdots \\ qc_{1}P_{k} \end{array}\begin{array}{c} qc_{2}P_{1}\\ \cdots \\ qc_{2}P_{k} \end{array}\begin{array}{c} \cdots \\ \cdots \\ \cdots \end{array}\begin{array}{c} qc_{k}P_{1}\\ \cdots \\ \left(1-q+qc_{k}\right)P_{k} \end{array}\right],$$

and the STM of a CSPBN with perturbation is given by:

(6) $$A=\left[\begin{array}{c} \left(1-q+qc_{1}\right){\tilde{P}}_{1}\\ \cdots \\ qc_{1}{\tilde{P}}_{k} \end{array}\begin{array}{c} qc_{2}{\tilde{P}}_{1}\\ \cdots \\ qc_{2}{\tilde{P}}_{k} \end{array}\begin{array}{c} \cdots \\ \cdots \\ \cdots \end{array}\begin{array}{c} qc_{k}{\tilde{P}}_{1}\\ \cdots \\ \left(1-q+qc_{k}\right){\tilde{P}}_{k} \end{array}\right],$$

where ***P***_*i*_ and ${\tilde{P}}_{i}$ denote the STMs for the Boolean network *i, i* ∈ {1, 2, …, *k*}, without and with perturbation respectively.

### Context-Sensitive Stochastic Boolean Networks (CSSBNs)

#### A CSSBN without perturbation

**Figure 1:**
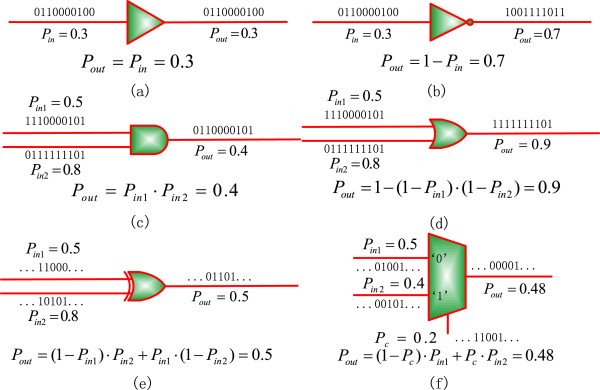
**Stochastic logic. (a)** A buffer (or an identity gate). **(b)** An inverter. **(c)** An AND gate. **(d)** An OR gate. **(e)** An XOR gate. **(f)** A multiplexer. Stochastic logic performs a probabilistic analysis by encoding probabilities into random binary bit streams as a proportional number of bits, for example, the mean number of 1’s in a binary sequence.

Because of the similarities between logic circuits and biological networks, digital circuits have been used to simulate genetic networks [30], as well as to determine the node vulnerability in cellular networks [31]. Stochastic logic has been demonstrated in several biological applications [32,33]. Stochastic computation implements probabilistic analysis using Boolean logic by encoding a real number or probability into a random binary bit sequence [34]. A probability is usually represented by a proportional number of 1’s in a stochastic sequence. The complement of a probability can be computed by an inverter and the multiplication of probabilities can be implemented by an AND gate for independent inputs. A multiplexer computes a weighted sum of its input probabilities, with the weights given by the selection inputs. Figure 1 shows an inverter (NOT), an AND gate, a buffer, an OR gate, an XOR gate and a multiplexer [24,35]. Due to stochastic fluctuations, the computational results by stochastic logic are not deterministic but probabilistic. However, this stochastic fluctuation can be reduced through the use of non-Bernoulli sequences of random permutations of fixed numbers of 1’s and 0’s as initial inputs, thus producing more accurate results than using Bernoulli sequences [35]. It has been shown that a probabilistic distribution of genes’ states can be accurately determined in a stochastic Boolean network (SBN) with a reasonably long stochastic sequence length [24].

In a CSPBN, the selection of a context is determined by the context switching probability. This probability indicates the likelihood to maintain the current context *i* or to select a new context from the *k* contexts (including the currently selected context *i*). Based on the present state and the selected context, a gene’s state is updated. If no perturbation occurs in an *n*-gene CSPBN with a switching probability of *q*, the transition probability from state (*s*, *y*) to (*r*, *x*) is given by [23]:

(7) $$p\left(\left(r,x\right)|\left(s,y\right)\right)=\left\{\begin{array}{c} f_{s,y,x}\left(1-q+qC_{s}\right)\\ f_{s,y,x}\left(qC_{r}\right) \end{array}\begin{array}{c} \mathrm{if}\;r=s\\ \mathrm{if}\;r\neq s \end{array}\right)$$

where

(8) $$f_{s,y,x}=\left\{\begin{array}{c} 1,\\ 0, \end{array}\begin{array}{c} \mathrm{if}\;y\;\mathrm{directly}\;\mathrm{transitions}\;\mathrm{to}\;x\;\mathrm{in}\;\mathrm{context}\;s\\ \mathrm{otherwise} \end{array}\right)$$

*s* and *r* denote the *s*th and *r*th contexts, *y* and *x* represent two gene activity profiles (GAPs, in decimal indices), and *C*_*s*_ and *C*_*r*_ indicate the probability of selecting the *s*th and *r*th contexts respectively.

**Figure 2:**
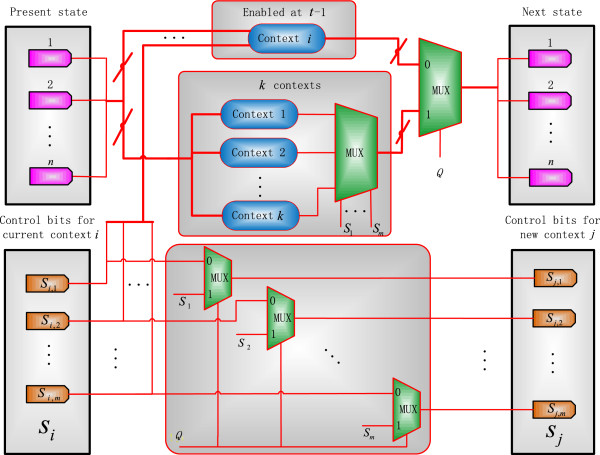
**A context-sensitive stochastic Boolean network (CSSBN) without perturbation (at time *t*).** The multiplexer (MUX) with control sequences *S*_1_ ~ *S*_*m*_ probabilistically determine the selection of a network function for context *i,* while the multiplexer with control sequence *Q* determines whether a switch of contexts occurs. The selection probabilities of the new context are computed by the 2-to-1 multiplexer (MUX) with the original and current context selection sequences as inputs and *Q* as the control sequence.

An SBN structure is proposed in [24] to implement an instantaneous PBN. For a CSPBN, a context-sensitive SBN (CSSBN) is constructed to consider the switching of contexts, as shown in Figure 2. In this CSSBN model, the probabilistic switching is implemented using a multiplexer in stochastic computation and the switching probability *q* is encoded as a random binary sequence *Q* that serves as the control sequence of a 2-to-1 multiplexer (MUX). If the *j*th bit in the sequence *Q* is 1, a new context will be selected for the next transition. Otherwise, the current context will remain. The selection probability of the new context is determined by the original and current context selection probabilities. As shown in the lower section of Figure 2, this process is implemented by 2-to-1 multiplexers with the original and current context selection sequences as inputs and *Q* as the control sequence. The selection probability of the new context is then obtained as encoded in the output stochastic sequences of the multiplexers. If *q =* 0, the CSSBN functions as a fixed Boolean Network (BN). If *q* = 1, the CSSBN is simplified to an instantaneous SBN.

If a switch does occur, the context selection process is implemented by another multiplexer for choosing one from the *k* contexts, according to predefined selection probabilities. As a network function is a combination of each gene’s predictor function, a combination of the *m* control sequences of *S*_1_ ~ *S*_*m*_ is used to encode the predefined selection probabilities; this in turn determines the selection probability of each new context. For a CSPBN with *n* genes, the CSSBN needs to be run for each of the 2^*n*^ input states and sequences need to be generated for the *m* control signals of the multiplexer.

For a switching probability of *q* in the proposed CSSBN, the currently selected context *i* remains at time *t* with a probability of *q* and switches to one of the *k* contexts with a probability of 1 - *q*. If the network transitions from a GAP *y* to *x* in the *s*th context (i.e., *f*_*s*,*y*,*x*_ = 1), then context *s* will remain with probability 1 - *q* + *qC*_*s*_ at the next time step. Otherwise, the network moves into a new context *r* with probability *qC*_*r*_. From this analysis, it can be seen that the CSSBN in Figure 2 computes the transition probability from state (*s*, *y*) to (*r*, *x*) as given by (7). This indicates that the proposed CSSBN model accurately implements the function of a CSPBN.

### A CSSBN with perturbation

**Figure 3:**
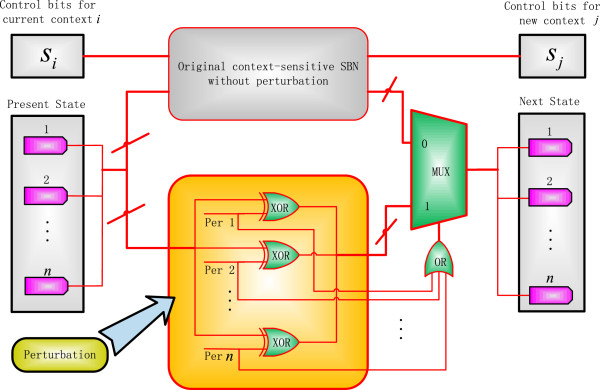
**A context-sensitive stochastic Boolean network (CSSBN) with perturbation.** A perturbation network is implemented by the XOR logic of the perturbation vector and the present state. The probability that either a new context works or a perturbation occurs is given by the output sequence of an *n*-input OR gate, which in turn determines the selection of a new context (without perturbation) or a perturbed network by a bus (or multiple-bit) multiplexer (MUX).

In a PBN with random perturbation, a gene may change its state with a probability *p* during a state transition. Following [12], the effect of perturbation is considered to flip a gene’s state. Assume in an *n*-gene CSPBN, the current GAP at time *t* is given by ***x*** = (*x*_1_, *x*_2_, …, *x*_*n*_) and ***γ*** is the perturbation vector, the GAP at *t* + 1, ***x′,*** is given by:

(9) $$\;x^{'}=\left\{\begin{array}{c} x⊕\gamma \\ f_{j}\left(x\right) \end{array}\begin{array}{c} \mathrm{with}\;a\;\mathrm{probability}\;\mathrm{of}\;1-{\left(1-p\right)}^{n}\\ \mathrm{with}\;a\;\mathrm{probability}\;\mathrm{of}\;{\left(1-p\right)}^{n}\; \end{array}\right)$$

where ⊕ is the addition modulo 2 and ***f***_*j*_(⋅) is the network function for the *j*th context at time *t.* To account for the effect of perturbation, a CSSBN with perturbation is constructed, as shown in Figure 3. In this CSSBN, XOR gates are used to implement the addition modulo 2 of the perturbation vector and the present state, while an *n*-input OR gate is used to compute the probability that a perturbation occurs. The output of the OR gate is then used as the control sequence of a bus (or multiple-bit) multiplexer to decide the selection of sequences with or without perturbation. If the output sequence of the OR gate contains all 0’s, which means that there is no perturbation, then the next state is given by the predictor functions in the currently selected context in the original CSSBN without perturbation; otherwise, the next state is determined by the perturbation effect. A stochastic analysis of the function of the CSSBN with perturbation shows that the next state of the network is given by:

(10) $$x^{'}=\left(x⊕\gamma \right)\cdot \left(1-{\left(1-p\right)}^{n}\right)+f_{j}\left(x\right)\cdot {\left(1-p\right)}^{n}$$

which is equivalent to (9). This indicates that a CSPBN with perturbation can be accurately implemented by a CSSBN with perturbation.

### Intervention in a CSSBN

In contrast to perturbation, gene intervention refers to the process of deliberately changing the states of some genes to guide a network into a desired state [12]. External stimuli are applied to a network to avoid undesirable states that might be associated with certain diseases. For an effective intervention, control policies are developed for different intervention strategies; as several genes may affect the state of the target gene, a single gene that is most influential on the network state is usually identified as the control gene, due to its simplistic biological implications [15]. The STM is then changed by an external intervention [15,21,22].

In a CSPBN, the context information is usually hidden and thus difficult to obtain in practice. However, gene expressions can readily be observed, so a GAP within all possible contexts is considered to be undesirable if the GAP is an undesirable state of the network [36]. Hence, gene intervention refers to changing the state of a network as represented by a GAP.

For an *n*-gene network, a vector ***s*** = (*s*_1_, *s*_2_, *s*_3_, …, *s*_*n*_) with *s*_*i*_ ∈ {0, 1} for any *i* ∈ {1, 2, …, *n*}, is defined as the control gene vector, that is, if *s*_*i*_ = 1, then gene *i* is selected as a control gene [37]. An intervention vector is defined as $u=\left(u_{1},u_{2},u_{3},\ldots ,u_{2^{n}}\right)$, *u*_*j*_ ∈ {0, 1} for any *j* ∈ {1, 2, …, 2^*n*^}, where *u*_*j*_ = 1 (or 0) indicates a flipping (or remaining) of the state of a control gene at GAP *j.* If gene *i* is selected to be a control gene, for example, then *s*_*i*_ = 1. The expression level of the control gene *i* is then determined by its current state and the status of ***u***. If *u*_*j*_ = 1, i.e., *s*_*i*_*u*_*j*_ = 1, the state of control gene *i* is flipped by the external intervention when GAP *j* emerges; otherwise, the state of control gene *i* remains unchanged and thus the current state is preserved in GAP *j*[38,39]. An intervention vector can be obtained by various methods; in this paper, a control policy using the steady state distribution (SSD) [40] is used to obtain ***u***.

The state of a network under intervention, i.e., a GAP, is determined by the control signal, the state prior to the intervention and the intervention vector. Let ${\hat{x}}_{t}$ and ***x***_*t*_ be the GAPs before and after intervention at time *t*; if the *g*th gene is the control gene (i.e., *s*_*g*_ = 1), the state of the network under intervention is given by [29]:

(11) $$x_{t}=\left({\hat{x}}_{t}⊕s_{g}\right)\cdot 1\left(u\left({\hat{x}}_{t}\right)=1\right)+{\hat{x}}_{t}\cdot 1\left(u\left({\hat{x}}_{t}\right)=0\right),$$

where ***s***_*g*_ is a vector of *n* bits with 0 at every bit except the *g*th bit. 1(∙) is an indicator function: $1\left(u\left({\hat{x}}_{t}\right)=1\right)=1$ if $u\left({\hat{x}}_{t}\right)=1$ and $1\left(u\left({\hat{x}}_{t}\right)=1\right)=0$ otherwise. When $u\left({\hat{x}}_{t}\right)=0$, the GAP ${\hat{x}}_{t}$ is maintained; otherwise, the GAP ${\hat{x}}_{t}$ is an undesirable state, for which the control bit is to be toggled, thereby improving the occurring probability of a desirable state.

**Figure 4:**
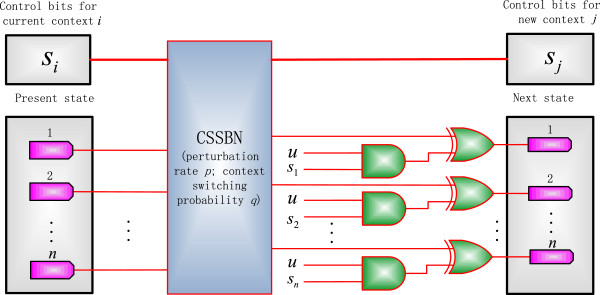
**A context-sensitive stochastic Boolean network (CSSBN) for external gene intervention. ***p*: perturbation rate; *q*: context switching probability. *s*_*i*_ indicates whether gene *i* is selected as a control gene and ***u*** is the derived intervention vector for all states or gene activity profiles (GAPs) of a network.

The CSSBN model under intervention is given in Figure 4. If gene *i* is selected as the control gene, i.e., *s*_*i*_ = 1, the output of the AND gate is determined by the state of ***u***. If state *j* is an undesirable state, i.e., *u*_*j*_ = 1, the state of the control gene *i* is toggled. This is implemented by an XOR gate: if *s*_*i*_*u*_*j*_ = 1, the state of gene *i,* given by the output of an XOR gate, is flipped at the state, or GAP, *j*; otherwise, the state is not changed by the intervention. From this analysis, the state of a network after intervention is given by:

(12) $$x_{t}=\left\{\begin{array}{c} {\hat{x}}_{t}⊕s_{i},\\ {\hat{x}}_{t}, \end{array}\begin{array}{c} \mathrm{if}\;u\left({\hat{x}}_{t}\right)=1\\ \mathrm{otherwise} \end{array}\right)$$

which is equivalent to (11). This indicates that the CSSBN model in Figure 4 accurately implements the process of external gene intervention.

### State transition matrix (STM) and steady state analysis

As discussed previously, the state of a CSPBN can be represented as ***S*** = (*context*, *x*) by considering the selected context and GAP information. Using a CSSBN, the STM can be obtained through the statistics encoded in the output sequences. Given an input state, each simulation of a CSSBN produces the transition probabilities from this input to all output states, i.e., the row in the STM for this input. For a CSPBN with *n* genes and *k* contexts, the CSSBN needs to be run for each of the 2^*n*^*k* input states and an *O*(*n*) number of sequences need to be generated for the control signals of the multiplexers. As in [24], a factor, *L*, is used to account for the computational overhead required by using a longer stochastic sequence. Therefore, a complexity of *O*(*nLk*2^*n*^) results for computing the STM of a context-sensitive network. In a CSSBN, the required minimum sequence length is typically on a polynomial order of the numbers of genes and contexts, as shown in the simulation results in Table 1. Since the number of possible BNs, *k*, generally increases exponentially with the number of predictor functions for each gene, the complexity of using a CSSBN to compute the STM, i.e., *O*(*nLk*2^*n*^), is smaller than *O*(*nk*^2^2^2*n*^) of an accurate analysis for a CSPBN [23]. As indicated by the shorter average run time in Table 1, this difference becomes significant for a network with a large number of gene states. In Table 1, the simulation results are also provided for CSSBNs with a single context, i.e., *k* = 1. In this case, the CSSBN degenerates into a deterministic BN, for which an analytical approach is more efficient, due to the use of random binary bit sequences in the stochastic approach. However, the CSSBN approach is more efficient in computing the STM for a gene network with a large number of states. See Additional file 1: The pseudocode for computing the STM of a CSSBN.

**Table 1:** Minimum sequence length and average run time in computing the state transition matrix (STM) for context-sensitive stochastic Boolean networks (CSSBNs)

***n***	***k***	***Num***	**CSSBN (Norm 2 = 0.05)**			**CSSBN (Norm 2 = 0.03)**			**CSPBN **[23]	
			***L*** **(bits)**	**Average time(s)**	**Standard deviation**	***L*** **(bits)**	**Average time(s)**	**Standard deviation**	**Average time(s)**	**Standard deviation**
2	1	4	40	0.00148	0.00048	100	0.00181	0.00048	0.00049	0.00046
2	4	16	900	0.00553	0.00167	2000	0.01116	0.00036	0.01248	0.00251
2	16	64	1000	0.64383	0.41941	6000	1.02666	0.10779	0.09593	0.00901
3	1	8	140	0.00446	0.00132	340	0.00812	0.00094	0.00157	0.00045
4	1	16	250	0.01438	0.00287	750	0.03685	0.00268	0.00443	0.00027
**4**	**16**	**256**	**8000**	**1.43115**	**0.01844**	**20000**	**3.29188**	**0.03830**	**1.66014**	**0.10349**
5	1	32	420	0.04602	0.00261	1200	0.12341	0.00314	0.01862	0.00214
**5**	**32**	**1024**	**8000**	**10.7359**	**0.01350**	**25000**	**35.5412**	**0.04335**	**24.0659**	**0.08390**
6	1	64	600	0.09993	0.00458	1700	0.33797	0.00197	0.06662	0.00705
**6**	**64**	**4096**	**24000**	**74.5058**	**1.20649**	**60000**	**195.793**	**4.86945**	**531.068**	**6.36186**
7	1	32	1000	0.52519	0.02762	2800	1.34503	0.06377	0.25873	0.00719
**7**	**16**	**2048**	**8000**	**18.8938**	**0.42939**	**25000**	**50.0857**	**0.27419**	**221.922**	**1.65582**
8	1	256	1400	1.45711	0.02798	3500	3.67661	0.16084	1.05147	0.01302
**8**	**4**	**1024**	**4000**	**5.50577**	**0.05167**	**12500**	**15.6728**	**0.11539**	**37.4102**	**2.20907**
9	1	512	2600	5.36480	0.07636	8000	16.2693	0.14106	4.06749	0.06899
**9**	**4**	**2048**	**8000**	**23.1989**	**0.40195**	**18000**	**45.9247**	**0.78522**	**72.4459**	**2.62472**
10	1	1024	4000	18.4761	0.12540	11000	49.2457	0.36526	17.5134	0.12540
**10**	**4**	**4096**	**10000**	**59.9416**	**0.41164**	**30000**	**162.686**	**1.13288**	**619.311**	**13.4098**

*n*: the number of genes; *k*: the number of contexts. Perturbation and context switch probabilities: *p* = 0.1 and *q* = 0.8 (for *k* > 1). *L*: the required minimum sequence length for a given accuracy measured by Norm 2. *Num*: the number of states for the CSSBN. The results that at least one case of the CSSBN study is more efficient than the CSPBN, are highlighted in bold.

In general, a (2^*n*^ ∙ *k*) × (2^*n*^ ∙ *k*) STM is required for an accurate CSPBN analysis [23] while a 2^*n*^ × 2^*n*^ STM is needed by an approximate method [20]. As the number of genes increases in a network, a matrix-based analysis becomes infeasible due to a significant increase in the required computational resources. The analysis of the SSD is even more challenging for a CSPBN. For a CSSBN, however, the STM can be accurately and efficiently computed. Furthermore, the SSD can be evaluated through an iterative simulation in the temporal domain (or the so-called time-frame expansion technique [24]). By this technique, an iterative structure of the CSSBN is used to simulate the temporal evolution of a GRN. The required number of iterations is determined by the number of state transitions before reaching a steady state.

Given an initial state distribution ***x***^(0)^, let ***x***^(*m*)^ and ***x***^(*m* + 1)^ be the state distributions after *m* and *m* + 1 transitions respectively. Assume that the STM of the network is given by ***A***, then the state transitions from ***x***^(*m*)^ to ***x***^(*m* + 1)^ are described by:

(13) $$x^{\left(m+1\right)}=x^{\left(m\right)}\cdot A$$

If || ***x***^(*m* + 1)^ - ***x***^(*m*)^||_∞_ is used to compute the maximum absolute value of the summed difference of each row in ***x***^(*m*)^ and ***x***^(*m* + 1)^, the condition for reaching a steady state is given by [18]:

(14) $${||x^{\left(m+1\right)}-x^{\left(m\right)}||}_{\infty }<\epsilon$$

where ϵ indicates a threshold for determining whether the steady state has been reached or not. If (14) is met, a network is considered to have reached a steady state, i.e., ***x***^(∞)^ = ***x***^(*m*)^; thus ***x***^(*m*)^ is considered as the stationary distribution. Alternatively, a reasonable number of network transitions (e.g. a few hundreds) can be run to obtain the SSD in practice. As shown in the Results and Discussion section, a time-frame extended simulation of CSSBNs is more efficient than an analytical approach while producing more accurate results than an approximate analysis. See Additional file 2: The pseudocode for computing the SSD of a CSSBN.

In a time-frame expanded CSSBN, random binary bit streams are generated for predictor function selection, gene perturbation and context switching probabilities. As in [24], non-Bernoulli sequences of random permutations of fixed numbers of 1’s and 0’s are used for encoding initial probabilities. These sequences then propagate through the CSSBNs and the statistics encoded in the output sequences are used to obtain the SSD. Compared to the use of Bernoulli sequences, as shown in [35], the use of the non-Bernoulli sequences produces more accurate results for a given sequence length or requires a shorter sequence length for a desired evaluation accuracy. As an efficient alternative to an STM-based analysis, the stochastic simulation of CSSBNs provides flexibility in achieving a tunable accuracy-efficiency tradeoff by using stochastic sequences of different lengths. Hence, the proposed CSSBN approach is potentially useful in the analysis of large GRNs.

## Results and discussion

### Simulation with a p53-Mdm2 Network

As a tumour suppressor gene, p53 plays an important role in preventing the development and progression of tumour cells [41,42]. In a p53 network, signaling pathways are triggered by external stimuli. For example, DNA damages activate pathways that involve the genes p53 and Mdm2. It has been shown that the expression level of the p53 protein is reversely related with that of the Mdm2 gene, which leads to an oscillatory behavior of the p53-Mdm2 network [25,43]. Various Boolean models have been developed to simulate the dynamics of a p53 network [44-46]. In this section, the two-gene probabilistic Boolean network (PBN) model developed in [24] for the p53-Mdm2 network is used to illustrate the applicability of context-sensitive stochastic Boolean networks (CSSBNs). This (instantaneous) PBN of the two genes p53 and Mdm2 consists of *V* = {*x*_1_, *x*_2_} and the predictor function sets $F_{1}=\left\{f_{1}^{\left(1\right)},f_{2}^{\left(1\right)},f_{3}^{\left(1\right)},f_{4}^{\left(1\right)}\right\}$ and $F_{2}=\left\{f_{1}^{\left(2\right)},f_{2}^{\left(2\right)},f_{3}^{\left(2\right)},f_{4}^{\left(2\right)}\right\}$. The truth table for the state transitions of this PBN is given as Table 2[24].

**Table 2:** **Truth table of a PBN for the p53-Mdm2 network: *x***_**1**_**,** ***x***_**2**_**are the present states of p53 and Mdm2**

***x***_**1**_ ***x***_**2**_	$f_{1}^{\left(1\right)}$	$f_{2}^{\left(1\right)}$	$f_{3}^{\left(1\right)}$	$f_{4}^{\left(1\right)}$	$f_{1}^{\left(2\right)}$	$f_{2}^{\left(2\right)}$	$f_{3}^{\left(2\right)}$	$f_{4}^{\left(2\right)}$
00	1	1	1	0	0	0	0	1
01	1	1	0	0	0	0	1	1
10	0	0	1	1	1	1	0	0
11	0	1	1	1	1	0	0	0
$\;c_{j}^{\left(i\right)}$	0.5	0.4	0.09	0.01	0.5	0.4	0.09	0.01

An internal entry indicates whether a logical 1 or 0 would result at the next state of a gene from a selected Boolean function. $c_{j}^{\left(i\right)}$ indicates the transition probability by a function $f_{j}^{\left(i\right)}$[24].

An internal entry indicates whether a logical 1 or 0 would result at the next state of a gene from a selected Boolean function. $\;c_{j}^{\left(i\right)}$ indicates the transition probability by a function $f_{j}^{\left(i\right)}$[24].

**Figure 5:**
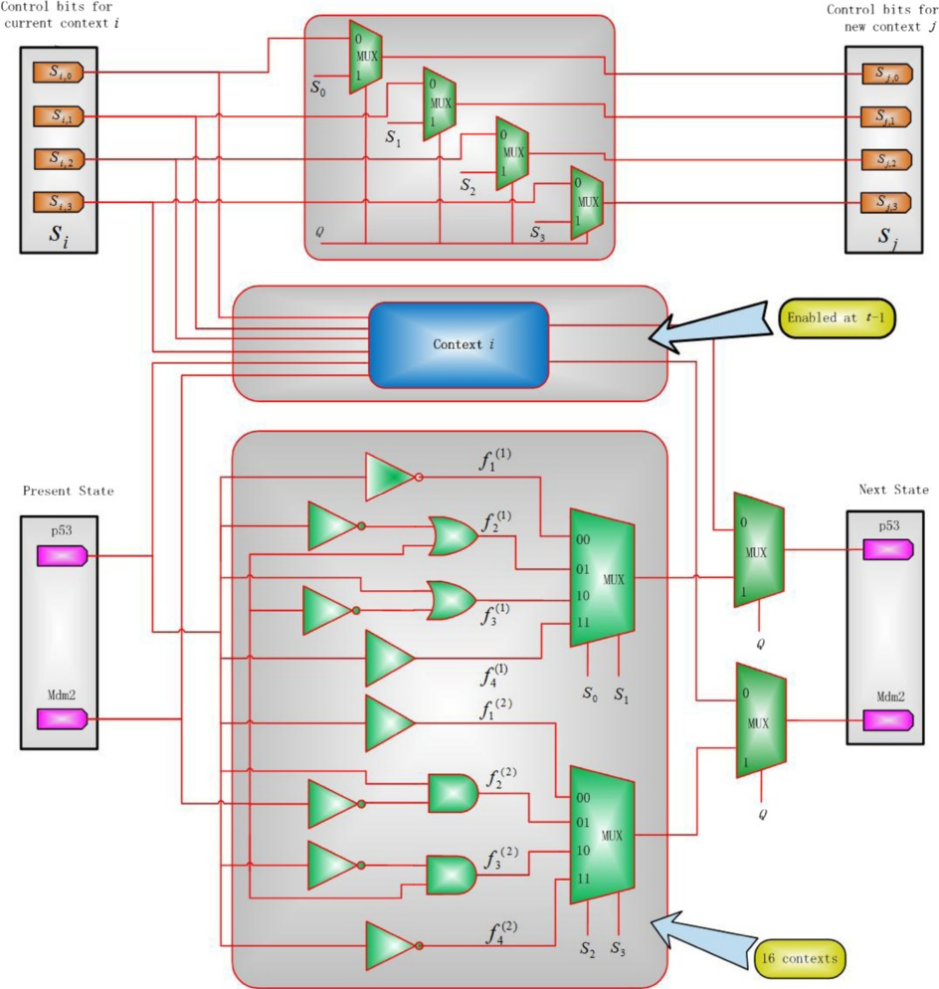
A context-sensitive stochastic Boolean network (CSSBN) for the p53-Mdm2 network.

In [24], a stochastic Boolean network (SBN) of the p53-Mdm2 network is proposed. Based on the general CSSBN model in Figure 2, a CSSBN for the p53-Mdm2 network is shown in Figure 5. In this model, a network function (or a Boolean network (BN)) is selected at time *t* - 1 for context *i* and at time *t*, the network remains at context *i* with a probability of *q* or switches to one of the *k* contexts (*k* = 16 for this network) with a probability of 1 - *q*. This context switching behavior is modeled by a 2-to-1 multiplexer (MUX) for each gene and the switching probability *q* is encoded in the control bit sequence *Q*. At the same time, the selection probabilities of the new context are determined by the 2-to-1 multiplexers with the original and current context selection sequences as inputs and *Q* as the control sequence, as shown in the upper section of Figure 5.

**Figure 6:**
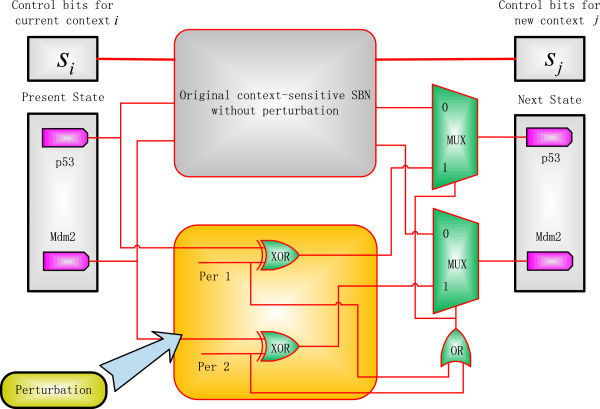
A context-sensitive stochastic Boolean network (CSSBN) with perturbation for the p53-Mdm2 network.

The context-sensitive p53-Mdm2 network has 2 genes and 16 contexts. If both context and gene activity profile (GAP) are considered, there are 2^2^ × 16 = 64 states for the p53-Mdm2 CSSBN. Given the transition probability for each predictor function (in Table 2), the probability for selecting each context can readily be computed for independent functions, as shown in Table 3.With random perturbation to the genes, a CSSBN with perturbation is constructed as shown in Figure 6. For this two-gene network, a two-input multiplexer is used for each gene to probabilistically select a perturbed state or the original CSSBN state without perturbation.

**Table 3:** The network function and selection probability for each context in the p53-Mdm2 network

**Context**	**S2S3, S0S1**	**Combination**	**Selection probability**	**Context**	**S2S3, S0S1**	**Combination**	**Selection probability**
1	00,00	$f_{1}^{\left(2\right)}f_{1}^{\left(1\right)}$	0.25	9	10,00	$f_{3}^{\left(2\right)}f_{1}^{\left(1\right)}$	0.045
2	00,01	$f_{1}^{\left(2\right)}f_{2}^{\left(1\right)}$	0.2	10	10,01	$f_{3}^{\left(2\right)}f_{2}^{\left(1\right)}$	0.036
3	00,10	$f_{1}^{\left(2\right)}f_{3}^{\left(1\right)}$	0.045	11	10,10	$f_{3}^{\left(2\right)}f_{3}^{\left(1\right)}$	0.0081
4	00,11	$f_{1}^{\left(2\right)}f_{4}^{\left(1\right)}$	0.005	12	10,11	$f_{3}^{\left(2\right)}f_{4}^{\left(1\right)}$	0.0009
5	01,00	$f_{2}^{\left(2\right)}f_{1}^{\left(1\right)}$	0.2	13	11,00	$f_{4}^{\left(2\right)}f_{1}^{\left(1\right)}$	0.005
6	01,01	$f_{2}^{\left(2\right)}f_{2}^{\left(1\right)}$	0.16	14	11,01	$f_{4}^{\left(2\right)}f_{2}^{\left(1\right)}$	0.004
7	01,10	$f_{2}^{\left(2\right)}f_{3}^{\left(1\right)}$	0.036	15	11,10	$f_{4}^{\left(2\right)}f_{3}^{\left(1\right)}$	0.0009
8	01,11	$f_{2}^{\left(2\right)}f_{4}^{\left(1\right)}$	0.004	16	11,11	$f_{4}^{\left(2\right)}f_{4}^{\left(1\right)}$	0.0001

The control bits to select a predictor function in Figure 5 are also listed. S0S1: the control bits for p53; S2S3: the control bits for Mdm2.

The CSSBN is then used to obtain the state transition matrix (STM) for the p53-Mdm2 network. See Additional file 3: The Matlab program that describes the structure of the CSSBN in Figure 6 and computes its STM for the p53-Mdm2 network with perturbation.

The norms || ⋅ ||_1_, || ⋅ ||_2_, and || ⋅ ||_∞_ are used to measure the differences of the STMs obtained for the CSSBN and CSPBN. || ⋅ ||_1_ and || ⋅ ||_∞_ indicate the maximum absolute values of the summed differences of the columns and rows respectively, while || ⋅ ||_2_ measures the average difference of all entries. Assume ***A***_*CSSBN*_ and ***A***_*CSPBN*_ are the obtained STMs for the CSSBN and CSPBN respectively. Let *Δ* ***A*** = ***A***_*CSSBN*_ - ***A***_*CSPBN*_; the norms of the differences of the computed matrices (||*Δ* ***A*** ||_1_, ||*Δ* ***A*** ||_2_ and ||*Δ* ***A*** ||_∞_) are then shown in Table 4 for different values of the switching probability *q* and perturbation rate *p*. The average run time is also shown for using the CSSBN.

**Table 4:** **Differences in the state transition matrices (STMs) obtained using the context-sensitive stochastic Boolean network (CSSBN) with perturbation, compared to the results by using the analytical context-sensitive probabilistic Boolean network (CSPBN) approach in**[23]

	***L*** **(bits)**	***q*** **= 1** ***p*** **= 0**	***q*** **= 0.99** ***p*** **= 0**	***q*** **= 0.5** ***p*** **= 0**	***q*** **= 0.8** ***p*** **= 0.01**
||*Δ* ***A*** ||_1_	1k	0.1820	0.2067	0.2320	0.3469
	10k	0.0754	0.0754	0.0605	0.0890
	100k	0.0291	0.0239	0.0306	0.0210
||*Δ* ***A*** ||_2_	1k	0.0642	0.0754	0.0706	0.1012
	10k	0.0258	0.0259	0.0207	0.0297
	100k	0.0101	0.0091	0.0097	0.0080
||*Δ* ***A*** ||_∞_	1k	0.0382	0.0413	0.0530	0.0888
	10k	0.0144	0.0145	0.0164	0.0249
	100k	0.0050	0.0055	0.0064	0.0074
Average time (s)	1k	0.0601	0.0547	0.0534	0.0623
	10k	0.3172	0.3180	0.3863	0.3186
	100k	2.8781	2.8531	2.8467	2.9226

The average run time of 20 simulations using the CSSBN is also shown for different perturbation rates, *p*, and context switching probabilities, *q. L*: sequence length.

As revealed in Table 4, the difference in the STMs computed using the CSSBN and an analytical CSPBN approach is significantly reduced by increasing the sequence length *L*. However, the inaccuracies, due to the inherent stochastic fluctuations in stochastic computation, are generally small and thus negligible. Hence, the proposed CSSBN model can be used to accurately and efficiently compute the STM of a CSPBN.

**Figure 7:**
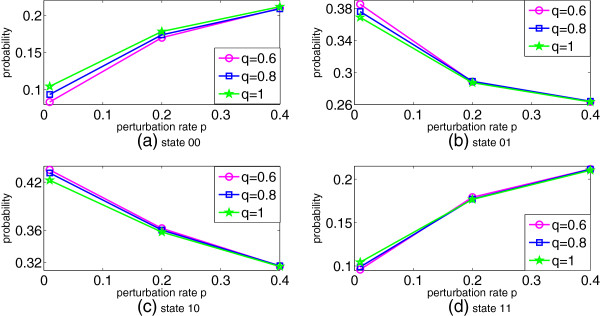
**The steady state distribution (SSD) of the p53-Mdm2 network for different perturbation rate, *p, *and context switching probability, *q.*** The steady-state probabilities are shown for **(a)** state 00, **(b)** state 01, **(c)** state 10, and **(d)** state 11. (*L* = 500k bits).

A steady state analysis is further performed on the proposed CSSBN. For the p53-Mdm2 network, there are four states or GAPs. The probability of each GAP is given by the sum of the probabilities for all contexts. The simulation results for the four GAPs with respect to different *p* and *q* values are shown in Figure 7 (using a sequence length of 500k bits).As can be seen in Figure 7, while the SSD is determined by both the perturbation and switching probabilities, the perturbation rate has a greater effect on the final distribution of the steady state compared to the switching probability. The SSD changes drastically with the increase of the perturbation rate, whereas the effect of context switching is rather limited for a given perturbation rate.

As *p*, *q* ∈ [0, 1], several *p* and *q* pairs are chosen for further analysis. The difference in the SSDs obtained by using the time-frame expanded CSSBN technique, the approximate [20] and accurate analysis [23] are shown in Table 5.

**Table 5:** **Differences in the steady state distributions (SSDs) computed using the context-sensitive stochastic Boolean network (CSSBN) model, compared to the results by using the approximate **[20]**and accurate analysis**[23]

			***p*** **= 0.01**	***p*** **= 0.1**	***p*** **= 0.3**
			***q*** **= 0.9**	***q*** **= 0.8**	***q*** **= 0.9**
||*Δ* ***SSD***_*AP* - *AC*_||_1_			0.0228	0.0214	0.0041
||*Δ* ***SSD***_*CSSBN* - *AC*_||_1_	*L* (bits)	10k	0.0094	0.0099	0.0073
		50k	0.0061	0.0060	0.0057
		100k	0.0047	0.0056	0.0040
||*Δ* ***SSD***_*AP* - *AC*_||_2_			0.0118	0.0118	0.0025
||*Δ* ***SSD***_*CSSBN* - *AC*_||_2_	*L* (bits)	10k	0.0055	0.0057	0.0041
		50k	0.0036	0.0036	0.0030
		100k	0.0028	0.0031	0.0025
||*Δ* ***SSD***_*AP* - *AC*_||_∞_			0.0074	0.0081	0.0020
||*Δ* ***SSD***_*CSSBN* - *AC*_||_∞_	*L* (bits)	10k	0.0047	0.0045	0.0027
		50k	0.0030	0.0029	0.0021
		100k	0.0020	0.0022	0.0017

*Δ* ***SSD***_*AP* - *AC*_: the difference between the results obtained by using the approximate and accurate analysis. *Δ* ***SSD***_*CSSBN* - *AC*_: the difference between the results obtained by using the CSSBN approach and the accurate analysis. *p*: perturbation rate, *q*: context switching probability, *L*: sequence length.

**Figure 8:**
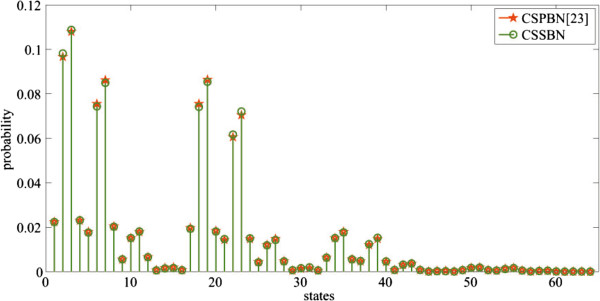
**Accuracy comparison of the proposed context-sensitive stochastic Boolean network (CSSBN) approach and the accurate analytical approach **[23]**for*p*** = **0.01,*q*** = **0.9 and*L*** = **10k bits.** The relative error is generally less than 0.2% for the CSSBN approach.

**Figure 9:**
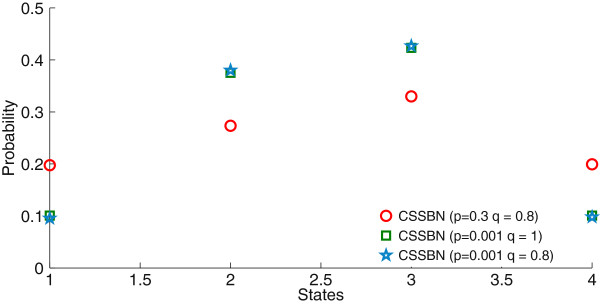
**Steady state distributions (SSDs) of the four states, or gene activity profiles (GAPs), obtained using the context-sensitive stochastic Boolean network (CSSBN) approach for different perturbation and switching probabilities.***L* = 10k bits.

As revealed in Table 5, the CSSBN approach can compute the SSD more accurately than the approximate analysis. In fact, the difference in the results between the CSSBN and the accurate analysis is negligible when reasonably long stochastic sequences are used. With the STM obtained for a CSSBN, an SSD is evaluated and the results are very accurate compared to those obtained using the accurate approach, as shown in Figure 8. A further analysis shows that the relative error is limited to less than approximately 0.2% for the CSSBN approach.The SSDs obtained using different methods, as well as the corresponding values of norms, are shown in Figure 9. It can be seen that the SSD can be accurately evaluated by the CSSBN model compared to the accurate analytical approach.

It has been shown that a PBN with perturbation evolves as an ergodic Markov chain [12]. For a larger perturbation probability *p,* there is an increased randomness in the network activities, thus the steady states of the network are more evenly distributed [23]. This can be seen in the simulation results in Figure 9. Figures 7 and 9 further show that context switching has little impact on the SSD of the network with perturbation. This is due to the fact that the context switching activity only affects the selection probability of a context, but not the predictor functions. Also shown in Figure 9 is that an oscillation of the states of p53 and Mdm2 exists, indicated by the higher probabilities of the network states (or GAPs) 01 and 10 compared with the other states. However, this two-state oscillation is less evident when the perturbation rate is significantly higher. On the other hand, a modest perturbation to the network only has a minor effect on the oscillatory behavior, confirming the stability and robustness of the p53-Mdm2 network in a dynamic environment [47].

### Experiments on a glioma network

**Figure 10:**
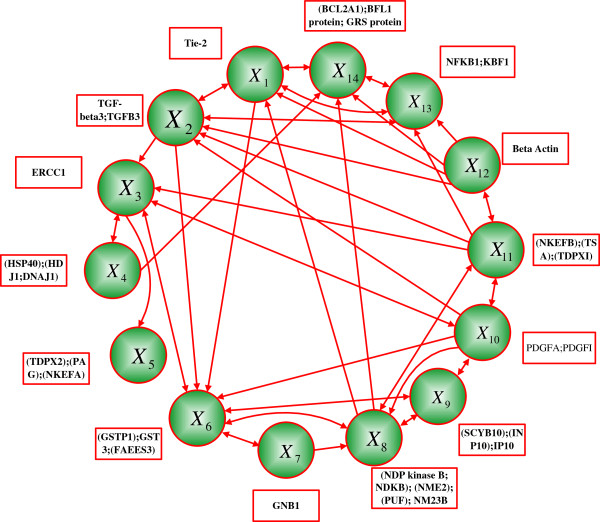
**A glioma network (adapted from **[18,20]**).**

A network in [16] obtained from a human glioma gene expression data set [48] is further used to illustrate the efficiency of the CSSBN model and the time-frame expansion technique. Based on this data set, a PBN was inferred by a method using the coefficients of determination (CODs) and an SSD analysis was performed in [18]. An approximate method was developed in [20] as an extension to estimate the SSD of a CSPBN with perturbation. The gene TOP2A was not considered in either study as it is an input gene with an in-degree of zero. In our study, the network setting is considered the same as in [18,20] with the gene TOP2A removed; this leads to a total of 2^14^ GAPs. Figure 10 shows a detailed structure of the considered glioma network with double (or single)-headed arrows indicating the bi (or uni)-directional relationships of gene pairs. The selection probabilities for the predictor functions are shown in Table 6[20] and the Boolean functions for each gene are obtained through an analysis of the data in [18].

**Table 6:** **Selection probabilities of the Boolean functions, *F***_***i***_**,** ***i*** **∈ {1, 2, …, 14}, for each gene in the glioma network in Figure**10[18,20]

***F***_**1**_	***F***_**2**_	***F***_**3**_	***F***_**4**_	***F***_**5**_	***F***_**6**_	***F***_**7**_
0.8560	0.2768	0.6759	1.0000	1.0000	0.0263	1.0000
0.1440	0.7232	0.3241			0.4983	
					0.4754	
***F***_**8**_	***F***_**9**_	***F***_**10**_	***F***_**11**_	***F***_**12**_	***F***_**13**_	***F***_**14**_
0.0857	1.0000	1.0000	0.8508	1.0000	0.8697	0.6004
0.9143			0.1492		0.1303	0.3996

In Table 6, each column for *F*_*i*_, *i* ∈ {1, 2, …, 14}, contains the selection probabilities for the Boolean functions of gene *i*, with the value in the *j*th row as the probability for $f_{j}^{\left(i\right)}$, thus the probabilities sum to 1 in each column. Table 6 also shows that six genes have only a single predictor function; only the state of gene 6 is determined by three functions, while the states of the other genes are determined by two predictor functions.

### Steady state analysis

**Figure 11:**
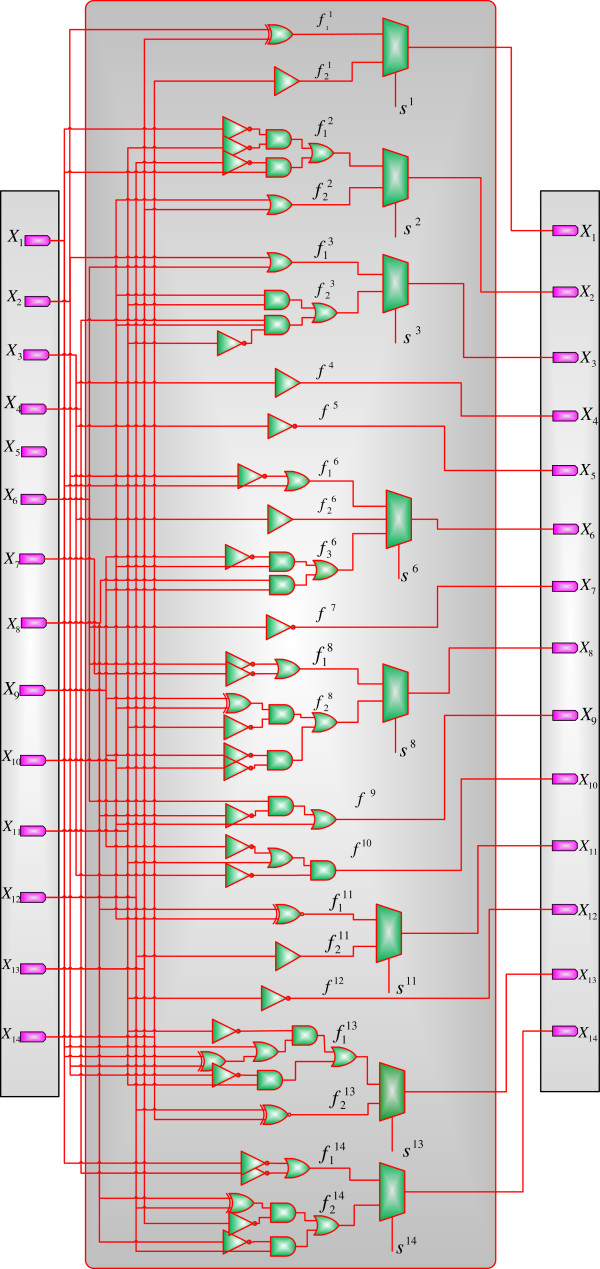
A stochastic Boolean network (SBN) for the glioma network as a basis for the context-sensitive SBN (CSSBN) model with 384 contexts.

For the 14-gene glioma network, there are a total of 384 contexts, as can be determined from Table 6. It requires an STM with 2^14^ × 384 = 6291456 columns and rows for an accurate analysis. This makes it infeasible to estimate the steady states of a CSPBN using a matrix-based analysis. The approximate analysis in [20] would require the computation of an STM of the size 2^14^ × 2^14^. Thus, it is difficult in general to use an analytical approach to evaluate a large network, due to the demanding computational resources required. However, a CSSBN model can be constructed for the glioma network; this CSSBN is based on the constituent SBN, as shown in Figure 11. With the CSSBN, the SSD can be estimated using the aforementioned time-frame expansion technique with a greatly reduced complexity. The obtained SSD is then compared with that obtained from the approximate analysis in [20]. In this paper, a network is considered to have reached a steady state if the discrepancy between two adjacent iterations is smaller than a required threshold ϵ (by (14)) or the number of simulation iterations has reached a maximum constant value. See Additional file 4: The Matlab program that evaluates the steady state distribution using the time-frame expanded CSSBN technique for the glioma network with context switching and perturbation.

**Figure 12:**
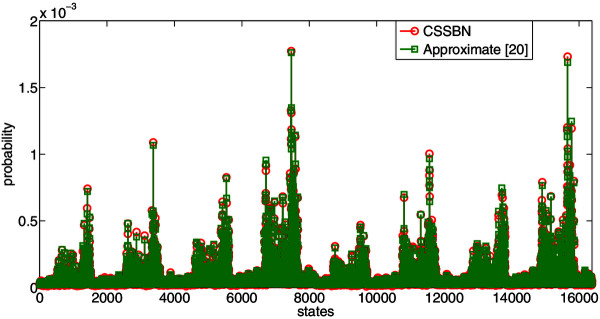
**The steady state distributions (SSDs) of the glioma network obtained using the CSSBN time-frame expansion technique and the approximate analysis **[20]**.***L* = 800k bits.

The state or GAP of the glioma network can be represented by a binary vector as (*x*_1_*, x*_2_*,* …*, x*_14_), *x*_*i*_ ∈ {0, 1} for *i* ∈ {1, 2, …, 14}, or its decimal index. For example, the state (01001100011011) can be represented as state 4892. The SSDs of the context-sensitive glioma network with all 16384 states, obtained using the CSSBN approach and the approximate analysis [20], are shown in Figure 12.

The norms of the differences of SSDs, obtained using the CSSBN with different sequence lengths and the approximate method in [20], are shown in Table 7.

**Table 7:** Norms of the differences in the computed steady state distributions (SSDs) and average run time for the glioma network

		***p*** **= 0.01** ***q*** **= 0.9**			***p*** **= 0.1** ***q*** **= 0.9**		
***L* (bits)**		**50k**	**200k**	**1M**	**50k**	**200k**	**1M**
||*Δ* ***SSD***_*AP* - *CSSBN*_||_1_		0.2314	0.1323	0.0861	0.4251	0.2104	0.0939
||*Δ* ***SSD***_*AP* - *CSSBN*_||_2_		0.0055	0.0032	0.0023	0.0045	0.0023	0.0010
||*Δ* ***SSD***_*AP* - *CSSBN*_||_∞_		0.0013	8.01 × 10^- 4^	4.13 × 10^- 4^	3.05 × 10^- 4^	2.16 × 10^- 4^	7.85 × 10^- 5^
Average time (s)	CSSBN	10.106	311.55	1377.5	10.069	315.65	1380.7
	Approximate	21421			21417		

A maximum of 300 iterations are used for obtaining the SSD. *p*: perturbation rate; *q*: context switching probability; *L*: sequence length. *Δ* ***SSD***_*AP* - *CSSBN*_: the difference between the SSDs obtained by the approximate analysis [20] and the context-sensitive stochastic Boolean network (CSSBN) approach.

As shown in Table 7, the CSSBN time-frame expansion technique efficiently evaluates the SSD of the glioma network and produces results comparable to those obtained by the approximate analysis [20]. Evaluation accuracy is further improved by using longer stochastic sequences with yet a shorter runtime compared to the approximate method. Since it is difficult, if not impossible, to compute the STM or SSD of a large GRN by using an accurate or approximate analysis, the CSSBN time-frame expansion approach provides an alternative means to accurately and efficiently estimate the SSD of a large network with a tunable accuracy by using stochastic sequences of different lengths.

### Intervention analysis

In an *n-*gene network, if gene *X*_*i*_ is the target gene, the states of all genes can be divided into a set of desirable states, D, and a set of undesirable states, U, by the expression level of the target gene [40]. As can be seen in Figure 10, gene *X*_14_, the (BCL2A1); BFL1 protein; GRS protein is one of the most influential genes that interacts closely with others, thus it is chosen as the target gene. The desirable and undesirable states are then separated by the expression level of *X*_14_. Assume that the inactivation of *X*_14_ is preferred; the cumulative probabilities of the desirable and undesirable states are given by $\sum {{{}_{x}}_{{}_{14}}}_{=0}\pi_{x}$ and $\sum {{{}_{x}}_{{}_{14}}}_{=1}\pi_{y}$, respectively, where *π*_*x*_ and *π*_*y*_ are elements in the desirable and undesirable SSDs respectively.

As discussed previously, a GRN can be intervened by applying external stimuli to minimize the likelihood of being in an undesirable state. Various methods have been proposed for deriving control vectors for an effective intervention [29,40]; in this study the SSD algorithm proposed in [40] is used to determine an intervention vector. For simplicity, 16 most significant contexts that account for a total selection probability of 57.27% are chosen from the total 384 contexts of the glioma network for an intervention analysis. For these 16 contexts, the expression levels of gene *X*_5_ and *X*_7_ have no effect on the states of other genes, so these two genes are removed; this yields a simplified 12-gene glioma network. The cumulative distributions of the desirable steady states of the context-sensitive 12-gene glioma network are shown in Table 8 for using a different gene as the control gene.

**Table 8:** **Cumulative distributions of the desirable states with a different gene selected as the control gene for the simplified 12-gene context-sensitive glioma network, with perturbation rate *p*** **= 0.001 and context switching probability** ***q*** **= 0.99**

**Gene**	***X***_**1**_	***X***_**2**_	***X***_**3**_	***X***_**4**_	***X***_**6**_	***X***_**8**_	***X***_**9**_	***X***_**10**_	***X***_**11**_	***X***_**12**_	***X***_**13**_
$\sum_{x_{14}=0}\pi_{x}$	70.91%	62.93%	52.61%	45.90%	48.98%	54.78%	53.76%	61.61%	57.23%	56.99%	61.78%

The cumulative probability of the desirable states with no intervention is 45.97%. Sequence length *L* = 800k bits.

When a different gene is selected as the control gene, the effect of intervention varies with respect to improving the probabilities of desirable states in the SSD. For the glioma network, as revealed in Table 8, gene X_1_, Tie-2, is the most effective for maximizing the percentage of the desirable states. The cumulative distribution of the desirable states is increased from 45.97% to 70.91% by an intervention using Tie-2 as the control gene. Also revealed in the table is that an intervention via gene *X*_4_, (HSP40); (HDJ1; DNAJ1), has nearly no impact on the distribution of the desirable states, as the percentage of the desirable states has not been changed much by the intervention (i.e., 45.90% vs. 45.97%). A modest improvement in the desirable state distribution is obtained by an intervention with another gene as the control gene. For any target gene, this process can be applied to find the most significant gene that maximizes the probabilities of desirable states.

**Figure 13:**
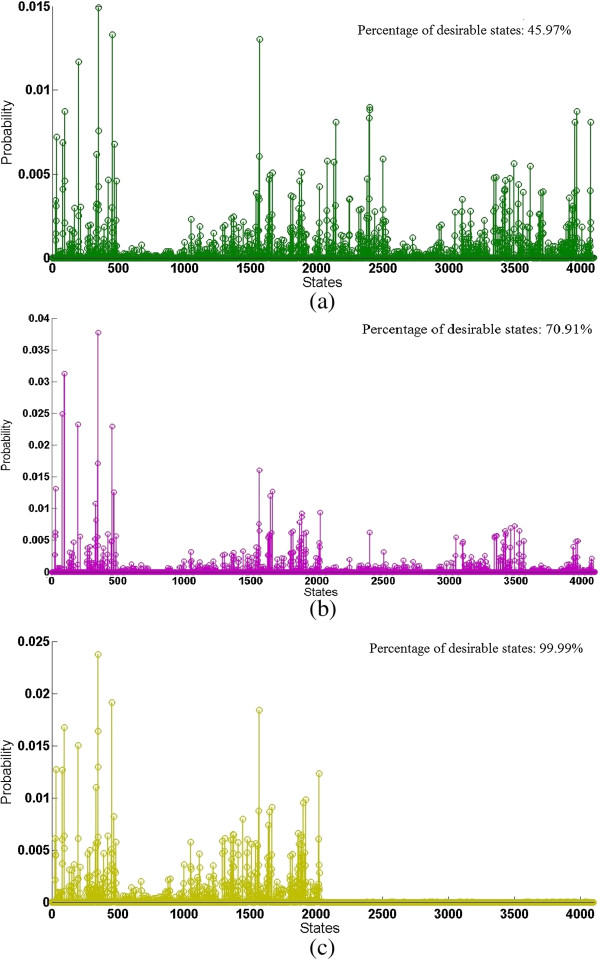
**Steady state distributions (SSDs) of the 12-gene context sensitive glioma network (*p*** **= 0.001,*q*** **= 0.99) obtained by: (a) no intervention, (b) an indirect intervention via gene*X***_**1**_**, Tie-2, and (c) a direct intervention via the target gene*X***_**14**_**, the (BCL2A1); BFL1 protein; GRS protein.**

The effects of intervention can also be seen in Figure 13, where three different scenarios are considered: (a) no intervention, (b) an indirect intervention via gene *X*_1_, i.e., Tie-2, and (c) a direct intervention via the target gene *X*_14_, the (BCL2A1); BFL1 protein; GRS protein. The cumulative probabilities of the desirable states are 45.97% for no intervention, 70.91% and 99.99% for the two intervention strategies. As revealed in these results, a direct intervention of the target gene is perhaps optimal for avoiding the undesirable states and almost certainly taking the network into a desirable state. However, when an intervention on the target gene is not possible, an intervention through Tie-2 is the most effective means to maximize the probability of the down-regulation of the target gene *X*_14_.

## Conclusion

Context-sensitive stochastic Boolean networks (CSSBNs) are proposed as an efficient approach to modeling the effects of gene perturbation and intervention in gene regulatory networks (GRNs). In a CSSBN, the state transition matrix can be accurately and efficiently computed with a complexity of *O*(*nLk*2^*n*^), where *n* is the number of genes in a context-sensitive probabilistic Boolean network (CSPBN), *k* is the number of contexts and *L* is a factor determined by the stochastic sequence length. This result is an improvement compared to the previous result of *O*(*nk*^2^2^2*n*^) for an accurate analysis. The use of non-Bernoulli stochastic sequences further increases computational efficiency and allows for a tunable tradeoff between accuracy and efficiency. A steady state analysis using a time-frame expansion technique has shown a significant speedup and produced more accurate results than an approximate analysis in the computation of the steady state distribution.

CSSBNs are constructed for the analysis of gene perturbation in a p53-Mdm2 network and gene intervention in a glioma network. Biologically meaningful insights are gained into the oscillatory dynamics of the p53-Mdm2 network in a context-switching environment with random gene perturbation. It has been shown that the steady state distribution (SSD) changes drastically with the increase of the perturbation rate, whereas the effect of context switching is rather limited for a given perturbation rate. Therefore, random gene perturbation may have a greater effect on the final distribution of the steady state compared to context switching activities. By predicting the SSD, the CSSBN approach can further evaluate the effectiveness of external gene intervention. A case study of the glioma network shows that the CSSBNs are useful in modeling the effects of gene perturbation and intervention in a complex context-sensitive GRN. This will eventually help drug discovery for an effective drug intervention therapy.

## Competing interests

The authors declare that they have no competing interests.

## Authors’ contributions

PZ carried out the GRN studies, performed the statistical analysis and drafted the manuscript. JL developed the initial models and participated in discussion. JH participated in the GRN studies, revised the manuscript and supervised the research. All authors read and approved the final manuscript.

## Supplementary Material

Additional file 1 The pseudocode for computing the state transition matrix (STM) of a context-sensitive stochastic Boolean network (CSSBN). Click here for file

Additional file 2 The pseudocode for computing the steady-state distribution (SSD) of a context-sensitive stochastic Boolean network (CSSBN). Click here for file

Additional file 3 **The Matlab program that describes the structure of the context-sensitive stochastic Boolean network (CSSBN) in Figure** 6**and computes its state transition matrix for the p53-Mdm2 network with perturbation.** Click here for file

Additional file 4 The Matlab program that evaluates the steady state distribution using the time-frame expanded context-sensitive stochastic Boolean network (CSSBN) technique for the glioma network with context switching and perturbation. Click here for file
