# Supplementary material for: Gene perturbation and intervention in context-sensitive stochastic Boolean networks
Source: BMC Syst Biol. 2014 May 21;8:60. doi: 10.1186/1752-0509-8-60 (PMC4062525; doi:10.1186/1752-0509-8-60)
Supplement: Additional file 1 — The pseudocode for computing the state transition matrix (STM) of a context-sensitive stochastic Boolean network (CSSBN). [file 1752-0509-8-60-S1.pdf]

Gene Perturbation and Intervention in Context-Sensitive Stochastic Boolean Networks

Peican Zhu, Jinghang Liang, Jie Han

Additional File 1

The pseudocode for computing the state transition matrix (STM) of a context-sensitive stochastic Boolean network (CSSBN)

For a CSSBN of n genes with k contexts, given the number of predictor functions of each gene, the selection probability of each function, the perturbation rate, p, and context switching probability, q, as well as the stochastic sequences generated for the aforementioned probabilities, the STM of the CSSBN can be obtained as follows.

```
for i = 1: 2^n
    for j = 1: k
        {
         Using the CSSBN to obtain the output stochastic sequences that denote the transition
         probabilities for input i and context j:
             {
              For context j, update the next state of genes for the present (input) gene state i;

              If gene perturbation is considered, then use the perturbation sub-network of the
              CSSBN with a multiplexer (MUX) to obtain the next gene state of the CSSBN
              with perturbation.

              Update the context, i.e., a new context is selected by determining the stochastic
              sequences that denote the selection probabilities of the new context.
              (This is done by 2-1 multiplexers with the original and current context selection
              sequences (probabilities) as inputs and the stochastic sequence denoting the
              context switching probability q as the control sequence.)
             }
         Determine the transition probability between the input state i and context j and every
         other state, from the obtained output stochastic sequences of the CSSBN.
        }
    end
end
```
