# Supplementary material for: Gene perturbation and intervention in context-sensitive stochastic Boolean networks
Source: BMC Syst Biol. 2014 May 21;8:60. doi: 10.1186/1752-0509-8-60 (PMC4062525; doi:10.1186/1752-0509-8-60)
Supplement: Additional file 2 — The pseudocode for computing the steady-state distribution (SSD) of a context-sensitive stochastic Boolean network (CSSBN). [file 1752-0509-8-60-S2.pdf]

Gene Perturbation and Intervention in Context-Sensitive Stochastic Boolean Networks

Peican Zhu, Jinghang Liang, Jie Han

Additional File 2

The pseudocode for computing the steady state distribution (SSD) of a context-sensitive stochastic Boolean network (CSSBN)

For a CSSBN of n genes with k contexts, given the number of predictor functions of each gene, the selection probability of each function, the perturbation rate, p, and context switching probability, q, as well as the stochastic sequences generated for the aforementioned probabilities, the SSD of the CSSBN can be obtained as follows.

Start with an initial state for each gene and an initial context, with a pre-determined threshold, ε, and a maximum iteration number, K.

for i = 1: K

{

Using the CSSBN to obtain the output stochastic sequences that denote the next state probabilities and the selection probabilities of new contexts:

{

For the present state and current context, update the next state of genes using the CSSBN with perturbation;

Update the context, i.e., a new context is selected by determining the stochastic sequences that denote the selection probabilities of the new context.

}

Determine the next state distribution from the obtained output stochastic sequences of the CSSBN with perturbation.

Compute the maximum absolute value of the summed difference between the present state distribution and the next state distribution, denoted by Δ.

If $\Delta < \varepsilon$, stop the execution; else, $i = i + 1$.

}

The final next state distribution is obtained as the SSD of the CSSBN with perturbation.

end
